# Meta-Analysis of the Core Aroma Components of Grape and Wine Aroma

**DOI:** 10.3389/fpls.2016.01472

**Published:** 2016-09-30

**Authors:** Tina Ilc, Danièle Werck-Reichhart, Nicolas Navrot

**Affiliations:** Institut de Biologie Moléculaire des Plantes, Centre National de la Recherche Scientifique, Université de StrasbourgStrasbourg, France

**Keywords:** volatiles, aroma, gas chromatography, profiling, wine, grapes

## Abstract

Wine aroma strongly influences wine quality, yet its composition and its evolution during the winemaking process are poorly understood. Volatile compounds that constitute wine aroma are traditionally divided into three classes according to their origin: grape, fermentation, and maturation aroma. We challenge this view with meta-analysis and review of grape and wine volatiles and their precursors from 82 profiling experiments. We compiled a list of 141 common grape and wine volatiles and quantitatively compared 43 of them. Our work offers insight into complex relationships between biosynthesis of aroma in grapes and the changes during the winemaking process. Monoterpenes are one of the largest and most researched wine aroma compounds. We show that their diversity in wines is mainly due to the oxidative metabolism of linalool in grapes. Furthermore, we demonstrate that most of the linalool produced in grapes is converted to these oxidized derivatives.

## Introduction

A crucial determinant of the wine quality is aroma, the composition of which is still not fully understood, due mainly to the interactions of the genetic factors with the environment. Yet this knowledge is necessary to ensure consistent production of high quality wines (Polášková et al., 2008). Wine aroma is a complex mixture of volatile organic compounds. These are small, non-polar molecules that readily enter the gas phase and reach our nasal cavity while we smell or drink a glass of wine. Different volatile organic compounds in wine span a large range of concentrations. While it is tempting to think that the more abundant compounds impact the aroma more than the trace compounds, it is not necessarily the case. Human nose can perceive some of the compounds at very low concentrations, whereas others remain undetected even at high levels. The strength of aroma of a particular compound is expressed as an odor detection threshold, which is the lowest concentration perceivable to the human smell. Compounds with low sensory thresholds are often responsible for the characteristic smell of a particular food (Dunkel et al., 2014).

Wine aroma compounds differ in their origin and evolution during the winemaking process. Many authors have classified wine aroma compounds into three categories based on their origin: grape (or varietal) aroma, fermentation aroma and aging aroma, also called the wine bouquet (Rapp and Mandery, 1986; Ebeler, 2001; Styger et al., 2011). However, these three classes are not so clear-cut: ultimately most of the aroma precursors (even the simple ones) originate from grapes and are in some way modified by the fermentation process or aging.

The grape aroma is synthesized in grape berries by a variety of enzymes, including terpene synthases, *O*-methyl transferases, carotenoid cleavage dioxygenases, cytochromes P450 and probably other not yet characterized enzymes. Genetic variation in aroma biosynthesis genes cause differences in aroma between grapevine varieties: an allelic variant of 1-deoxy-D-xylulose-5-phosphate synthase, a terpenoid biosynthetic gene, causes accumulation of terpenoids in Muscat and Gewurztraminer grapes. An abundance of terpenoids gives these wines a distinct floral aroma (Battilana et al., 2009, 2011; Duchêne et al., 2009). In another example, differential expression of an *O*-methyl transferase gene results in higher production of methoxypyrazines, compounds evoking the typical capsicum aroma in Sauvignon wines (Guillaumie et al., 2013). The genetic factors underlying the aroma typicity of all other grapevine varieties—in Europe alone over 2000 varieties have been described (Lacombe et al., 2011)—remain unexplored.

Grape berries store most of the volatiles they produce as glycosides (Strauss et al., 1988). Possible functions of glycosylation are sequestration, detoxification and decrease of volatility and reactivity (Hjelmeland and Ebeler, 2014). Since glycosides are not volatile, they do not directly contribute to wine aroma. They do, however, affect the aroma indirectly: they form a precursor pool from which volatile aglycones can be released during yeast and malolactic fermentation, during vinification by addition of exogenous glycosidases, during wine aging owing to its low pH (Maicas and Mateo, 2005) and, as demonstrated recently, by enzymatic hydrolysis in the mouth, catalyzed by the enzymes in the saliva (Mayr et al., 2014). Aroma glycosides in grapes have either one or two sugar moieties attached to the aglycone (forming mono- or diglycosides, respectively). The first sugar moiety, directly attached to the aglycone, is in all cases glucose. The majority of glycosides (at least in the case of terpenoids) are present in the form of diglycosides, in which a second sugar—arabinose, apiose or rhamnose—is attached to the glucose (Maicas and Mateo, 2005). The diglycosides cannot be hydrolyzed by a β-glucosidase, and require other glycosidase enzymes to release the volatile aglycone (Gunata et al., 1988).

The winemaking process profoundly influences the wine aroma development. Processes contributing are the hydrolysis of glycosides present in the must and the production, particularly, of alcohols and esters, by the yeast (*Saccharomyces cerevisiae*) itself. Different yeast strains can produce remarkably different aroma profiles (Romano et al., 2003). After alcoholic fermentation wines are sometimes subjected to malolactic fermentation by *Oenococcus oeni*, the principal role of which is to reduce tartness or acidity of wine by converting malic acid to lactic acid. These bacteria can also alter the composition of aroma by, for example, promoting deglycosylation (Ugliano and Moio, 2006).

The evolution of wine aroma continues after the fermentation process. In addition to above-mentioned acid hydrolysis of aroma glycosides during wine aging, low pH can cause other important chemical changes. Williams et al. (1980b) postulated that some hydroxylated linalool derivatives undergo cyclisation or other rearrangements at low pH. In addition, during storage in oak barrels, compounds from the wood are extracted to the wine and influence its taste and aroma.

Standardized sample preparation (Gunata et al., 1985; Voirin et al., 1992) and analytic procedures are routinely performed for evaluation of grape and wine volatiles and their precursors. Grape juices or wines are extracted to a non-ionic solid phase, free volatiles are eluted by a non-polar solvent (pentane or a mixture of pentane and dichloromethane), and glycosylated volatiles are eluted by a more polar solvent (ethyl acetate or methanol). The free fraction can then be directly analyzed by gas chromatography. The bound fraction is enzymatically hydrolyzed before the analysis. Gas chromatography allows for simultaneous analysis of hundreds of volatiles with good resolution of structurally similar molecules. Furthermore, coupling to a mass spectrometer enables reliable identification of compounds by searching mass spectra databases even when analytical standards are not available.

Many research laboratories used this method to investigate how aroma is influenced by grape variety, grape ripening, environment and different winegrowing and winemaking techniques. While these studies have without doubt addressed some of these important questions, a comprehensive picture of wine aroma integrating these valuable data has not been painted yet. We collected, curated and analyzed these data to answer the following questions. What are the components of grape and wine aroma? At which concentrations are they present? To what extent are they glycosylated? We compared these parameters between grapes and wines. We then compared these data to the existing knowledge on the origin and evolution of aroma components with a particular focus on their biosynthesis. This meta-analysis adds to our understanding of wine aroma composition and development.

## Materials and Methods

### Data Collection

We collected published datasets on free and glycosylated grape and wine volatiles. We used search term “grape aroma profiling free glycosylated” on Google Scholar and reviewed citations of included publications. We reviewed 104 publications that reported profiling of grapes or wines. Foutry-five of them were excluded because they only contained information on free volatiles, and 8 because they only contained information on glycosylated volatiles. To ensure the compounds were annotated correctly, only publications that contained information of Kovats retention index were included. Further 31 publications were therefore excluded, which resulted in 19 selected publications (**Table 1**). Publications or samples that described *Vitis* species other than *Vitis vinifera* subsp. *vinifera* were excluded from all quantitative analysis, but used for validation of retention indices.

**Table 1:** Publications included in the analysis.

Reference	Sample type	Number of samples	Genotypes/Varieties
Yilmaztekin et al., 2015	Wine	1	Karaoglan
Sánchez-Palomo et al., 2015	Wine	5	Verdejo
Selli et al., 2006	Wine	2	Narince
Toci et al., 2012	Wine	2	Negroamaro
Oliveira et al., 2008	Wine	5	Lourerio, Alvarinho
Becatti et al., 2014	Wine	1	Sangiovese
Gómez García-Carpintero et al., 2011a	Wine	5	Bobal
Gómez García-Carpintero et al., 2011b	Wine	5	Moravia Agria
Gómez García-Carpintero et al., 2012	Wine	3	Moravia Dulce, Rojal, Tortosi
Boido et al., 2003	Grape	1	Tannat
Ghaste et al., 2015	Grape	6	F3P30, IASMA ECO3, F3P63, Riesling, Gewürztraminer, Moscato Rosa^∗^
Bureau et al., 2000	Grape	2	Muscat of Frontignan
Canosa et al., 2011	Grape	5	Mencía, Espadeiro, Caíño Redondo, Pedral, Sousón
Fenoll et al., 2009	Grape	2	Muscat Hamburg
Genisheva and Oliveira, 2009	Grape	14	Alvarinho, Arinto, Avesso, Azal, Batoca, Lourerio, Trajadura
Ugliano and Moio, 2008	Grape	1	Fiano
Vrhovsek et al., 2014	Grape	5	Sauvignon Blanc, Chardonnay, Gewürztraminer, Grüner Veltliner, Sangiovese
Hellín et al., 2010	Grape	2	Superior Seedless
Lopez-Tammames et al., 1997	Grape	15	Albarinho, Listan, Treixadura, Greanche, Parellada, Tempranillo, Viura, Xarel⋅lo
Baek and Cadwallader, 1999	Grape	(3)	*Vitis rotundifolia* (Muscadine)^∗∗^

*^∗^Non-vinifera genotypes (Nero, Isabella, *Vitis arizonica* and *Vitis cinerea*) were excluded from the quantitative analysis. ^∗∗^Excluded from the quantitative analysis.*

### Data Curation

Molecule names and their Kovats retention indices were aggregated in one file and manually checked for duplicates (synonyms), which resulted in 385 unique molecules. They were assigned to one of the 16 classes of volatiles (aliphatic alcohol, ketone, aldehyde, ester, or acid, monoterpene, norisoprenoid, sulfur-containing, phenol, aromatic alcohol, ketone, aldehyde, ester, or acid, nitrogen-containing or other). Two hundred and forty-four unique molecules appeared in less than three publications and were excluded from further analysis because of insufficient information. Kovats retention indices of the remaining 141 compounds were compared and in the cases where standard deviation of the retention indices exceeded 50, outliers were excluded (annotation of a compound was considered incorrect in this particular publication and was excluded from the dataset).

We paid particular attention to geometric isomers of some compounds, which elute close to each other and have similar or identical mass spectra and are therefore often misannotated. Such pairs of compounds are: pyranic linalool oxides, furanic linalool oxides and 8-hydroxylinalools. In case of pyranic linalool oxides, the two isomers were incorrectly annotated in the first paper reporting their structure (Felix et al., 1963). This error was identified and corrected later (Kreis et al., 1996), but some authors continued to use the incorrect annotation. All the data were curated so that the first eluted furanoid linalool oxide was re-annotated as *trans* isomer, and the second one as *cis*. Similarly, the first eluted pyranoid linalool oxide was annotated as *trans* and the second one as *cis* (Luan et al., 2006b). Retention indices of (*Z*) and (*E*)-8-hydroxylinalool were first reported in (Winterhalter et al., 1986) and the author claimed the NMR spectra were in agreement with previously published data. However, in a subsequent publication (Chassagne et al., 1999), which is also cited in the widely used database of volatiles Pherobase^1^, the retention indices of the two compounds are inverted even if the author received standards from the author of (Winterhalter et al., 1986). Recent publication (Hofer et al., 2014) confirms the correct elution order is (*Z*)-, followed by (*E*)-8-hydroxylinalool, as reported in the original publication (Winterhalter et al., 1986).

### Statistical Analysis

Statistical analysis was performed using the R software version 3.0.2 (R Development Core Team, 2011). For quantitative analysis 43 molecules with more than 30 data points were selected from the pool of 141 validated compounds. Concentrations of these compounds in free and glycoside bound forms were then extracted from the 20 publications. If a compound was only detected in the free fraction, it was assigned a concentration of 0 in the glycosylated fraction, and vice versa. Labels “not detectable”, “not quantifiable” and “trace” were all converted to zero concentration. Concentrations of free and bound form of each molecule were then added to get the “total concentration”. “Fraction glycosylated” was calculated by dividing the concentration of glycosylated compound with the total concentration.

Concentration units were not the same in all studies. All concentrations in wines were reported in μg L^-1^, some concentrations in grapes were reported in μg L^-1^ and some in μg kg^-1^. In addition, grape juice and wine have a different density, so the concentrations are not directly comparable, but since we compared them on a logarithmic scale we considered these differences negligible and did not adjust for them.

Log-transformed concentrations were compared using a two-sided *t*-test. Only molecules with 5 or more data points in each category (grape and wine) were included. Glycosylated fractions were compared using Mann-Whitney–Wilcoxon *u* test. Correlations were calculated on total concentrations (free + glycosylated) for compounds with more than 40 complete observations. Linear models for linalool derivatives were calculated on log total molar concentrations (free + glycosylated) with non-zero values. Linalool derivatives with low R^2^ value (<0.45) were excluded from the graph [hotrienol, (*cis*)-linalool oxide (pyranoid)]. Data, residuals normality and leverage were visually evaluated for each model.

## Results and Discussion

### Composition of Wine Aroma

**FIGURE 1:**
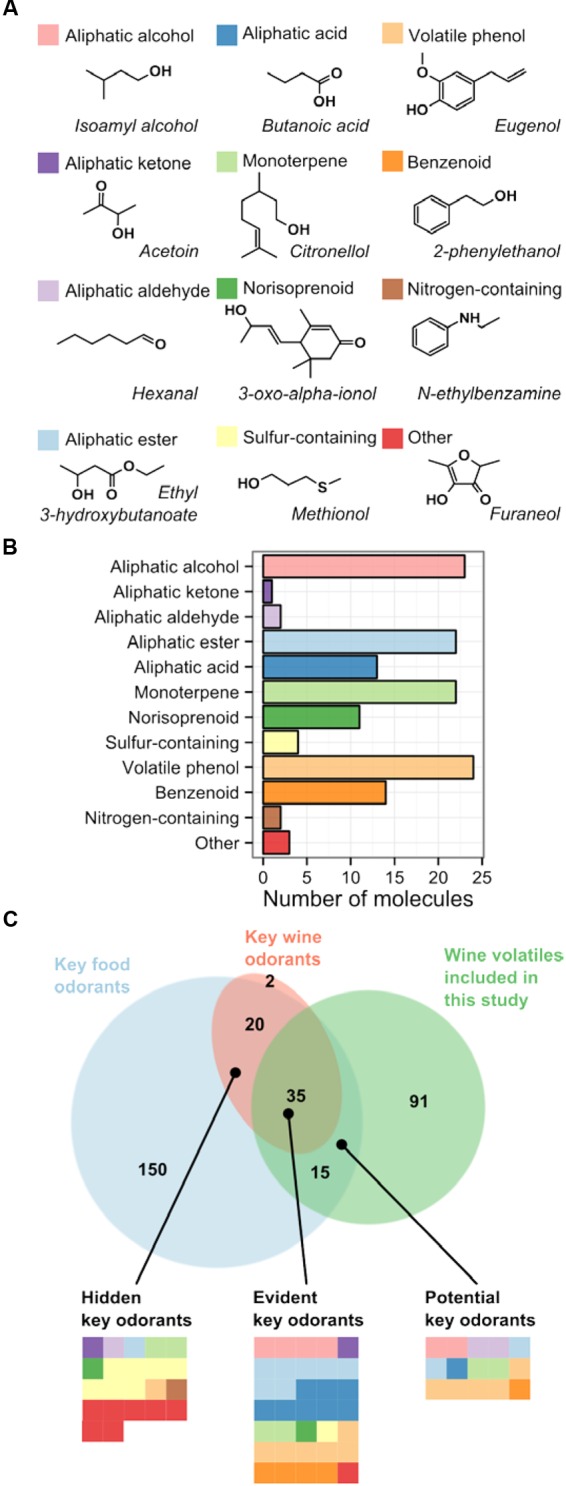
**Classes of grape and wine volatile compounds evaluated in this study. (A)** Classes of volatiles with examples. **(B)** Number of different molecules from each class that were included in this study. **(C)** Euler diagram of wine volatiles included in this study and key food and wine odorants, identified in two other meta-analyses. Two other meta-analyses identified volatile compounds with the strongest impact on aroma of food (Dunkel et al., 2014) and wine (Francis and Newton, 2005). While the overlap between the three studies is sizeable, many key odorants are not detected in volatile profiling experiments. Each tile represents one volatile molecule and its color corresponds to the molecular class.

Analysis of 19 publications describing grape or wine aroma revealed 385 different volatile organic compounds. More than half of them were only identified in one or two studies, and were eliminated from further analysis to account for the possibility of incorrect identification. Some of the eliminated compounds might be important varietal compounds, but this study focuses on the similarities, not the differences between the varieties. The remaining 141 validated volatile compounds (**Table 2**) were assigned to one of the 12 classes based on their chemical structure and biosynthetic origin. Authors of most reviews make a clear distinction between grape and fermentation derived wine aroma (Ebeler, 2001; Dunlevy et al., 2009; Styger et al., 2011; Robinson et al., 2014) and therefore use a different classification of wine aroma components. Classes defined in this study are based on classifications from other authors, while trying to contain all the molecules and to minimize the overlap between classes. The most represented classes (with respect to the number of validated compounds) were aliphatic alcohols, aliphatic esters, monoterpenes and volatile phenols (**Figure 1**).

**Table 2:** List of grape and wine volatiles reported in at least three studies with mean retention indices.

Compound	RI	Compound	RI	Compound	RI
**Aliphatic alcohols**					
Methanol	879	2-heptanol	1318	(*E*)-2-hexen-1-ol	1408
1-propanol	1045	4-methyl-1-pentanol	1319	(*Z*)-2-hexen-1-ol	1414
2-methyl-propanol (isobutanol)	1085	(*Z*)-2-penten-1-ol	1321	1-octen-3-ol	1452
1-butanol	1146	3-methyl-1-pentanol	1326	1-heptanol	1460
2-methyl-1-butanol	1212	1-hexanol	1357	2-ethyl-1-hexanol	1497
3-methyl-1-butanol (isoamyl alcohol)	1213	(*E*)-3-hexen-1-ol	1371	2,3-butanediol	1547
1-pentanol	1249	3-ethoxy-1-propanol	1377	1-octanol	1561
3-methyl-3-buten-1-ol	1252	(*Z*)-3-hexen-1-ol	1387		
**Aliphatic ketones**					
3-hydroxy-2-butanone (acetoin)	1284				
**Aliphatic aldehydes**					
Hexanal	1095	(*E*)-2-hexenal	1223		
**Aliphatic esters**					
Ethyl acetate	836	Ethyl lactate	1336	Diethyl succinate	1688
Isobutyl acetate	1012	Ethyl octanoate	1437	Ethyl methyl succinate	1743
Ethyl butanoate	1055	2-hydroxy 2-methylpropyl butyrate	1461	Methyl 4-hydroxybutyrate	1783
Ethyl 3-methylbutanoate	1070	Ethyl 3-hydroxybutanoate	1509	Ethyl 4-hydroxybutyrate	1822
Butyl acetate	1070	Ethyl lactate	1336	Ethyl dodecanoate	1852
Isoamyl acetate	1134	Diethyl malonate	1597	Diethyl malate	2057
Ethyl hexanoate	1218	Methyl decanoate	1629	Ethyl succinate	2359
Hexyl acetate	1285	Ethyl decanoate	1650	Ethyl 4-hydroxybutyrate	1822
**Aliphatic acids**					
Acetic acid	1440	Pentanoic acid	1705	Decanoic acid	2293
Propanoic acid	1524	Hexanoic acid	1835	Dodecanoic acid	2447
2-methylpropanoic acid (isobutyric acid)	1579	(*E*)-2-hexenoic acid	1942	Tetradecanoic acid (myristic acid)	2669
Butanoic acid (butyric acid)	1617	Octanoic acid	2055		
Isovaleric acid	1659	Nonanoic acid	2142		
**Monoterpenes**					
Limonene	1202	Citral/ (*E*)-geranial	1743	6-hydroxylinalool (diendiol II)	2152
*trans*-linalool oxide (F)	1447	Citronellol	1764	8-hydroxy-6,7-dihydrolinalool	2217
*cis*-linalool oxide (F)	1476	*cis*-linalool oxide (P)	1766	(*Z*)-8-hydroxylinalool	2274
Linalool	1547	Nerol	1811	(*E*)-8-hydroxylinalool	2304
Terpinen-4-ol	1599	Geraniol	1846	Geranic acid	2317
Hotrienol	1624	exo-2-hydroxy-1,8-cineole	1851	*p*-menth-1-ene-7,8-diol	2502
alpha-terpineol	1697	7-hydroxylinalool (diendiol I)	1950		
*trans*-linalool oxide (P)	1727	7-hydroxy-6,7-dihydrolinalool	1988		
**Norisoprenoids**					
4-oxo-isophorone	1703	3,4-dihydro-3-oxo-actinidol II	2420	3-hydroxy-7,8-dihydro-beta-ionol	2684
β-damascenone	1804	3-hydroxy-β-damascone	2553	3-hydroxy-7,8-dehydro-β-ionol	2732
3,4-dihydro-3-oxo-actinidol I	2382	Dehydrovomifoliol	2554	Vomifoliol	3148
3,4-dihydro-3-oxo-actinidol III	2412	3-oxo-alpha-ionol	2617		
**Sulfur-containing volatiles**					
3(2H)-2-methyldihydro-thiophenone	1505	3-methylthiopropanoic acid	1757	1,2-benzothiazole	
3-(methylthio)-1-propanol (methionol)	1719				
**Volatile phenols**					
Methyl salicylate	1779	Syringol (2,6-dimethoxyphenol)	2243	Ethyl vanillate	2676
Guaiacol (2-methoxyphenol)	1869	Isoeugenol	2302	3,4-dimethoxyphenol	2756
Phenol	1982	Methyl salicylate	1779	Zingerone	2796
4-ethylguaiacol	2055	Guaiacol (2-methoxyphenol)	1869	Tyrosol	3008
4-methyl phenol (p-cresol)	2087	4-methoxyphenylethyl alcohol	2302	Methyl vanillyl eter	3030
3-methyl phenol (m-cresol)	2119	4-vinylphenol	2388	3,4,5-trimethoxyphenol	3032
Eugenol	2181	(*E*)-4-allylsyringol	2424	4-methoxyphenylethyl alcohol	2302
4-ethylphenol	2187	Vanillin	2550	4-vinylphenol	2388
*p*-vinylguaiacol	2200	Methyl vanillate	2568		
4-hydroxy-2-methyl acetophenone	2212	Acetovanillone (Apocynin)	2654		
**Benzenoids**			
Benzaldehyde	1519	Ethyl benzene acetate	1782	Ethyl cinnamate	2286
Phenylacetaldehyde	1639	2-phenyl ethyl acetate	1809	Benzoic acid	2391
Acetophenone	1667	Benzyl alcohol	1883	Benzylacetic acid	2502
Ethylbenzaldehyde	1728	2-phenylethanol	1913	Cinnamic acid	3045
Benzyl acetate	1735	Benzenepropanol	2037		
**Nitrogen-containing volatiles**			
*N*-ethyl-benzamine	1750	*N*-(2-phenylethyl)-acetamide	2563		
**Other volatiles**			
gamma-butyrolactone	1628	Furaneol	2023	2,3-dihydrobenzofuran	2377

Not all volatiles contribute to the aroma equally. Their impact depends on their concentration, as well as aroma intensity. The latter is usually expressed as the odor detection threshold, which is the lowest concentration of a particular compound that can be detected by the human smell. In a recent meta-analysis, Dunkel et al. (2014) compiled a list of food volatiles occurring at concentrations exceeding their odor detection threshold, and named them key food odorants. Somehow surprisingly, a total of only 220 key food odorants are responsible for the aroma of most of the foods and beverages we consume, and among those fewer than 40 contribute to aroma of an individual food item. Another meta-analysis identified 57 key wine odorants, which were identified as wine volatiles with concentrations above their odor detection threshold. All but 2 of these volatiles are also included among the 220 key food odorants. Only 60% (*N* = 35) of those key wine odorants overlap with our set of common wine volatiles, compiled from non-targeted profiling experiments (**Figure 1**). We named that group “evident” key odorants. The other 40% (*N* = 22) of key wine odorants are overlooked in non-targeted volatile profiling experiments, presumably because of their low concentrations. We named that group of volatiles “hidden” key odorants. The two largest groups within the hidden key odorants are sulfur-containing volatiles and fatty acid lactones (Supplementary Table S1). Several sulfanyl (or mercapto) alcohols were identified as important varietal compounds in Sauvignon Blanc and many other varieties, but their low abundance requires the use of targeted analytical methods, such as stable isotope dilution analysis (SIDA) for their quantification (Roland et al., 2011) and were therefore not among the volatiles included in this study. Even among monoterpenes, one of the largest classes in our study, two odor-active molecules (*cis*-rose oxide and wine lactone) are not detected in non-targeted volatile profiling experiments. Conversely, the high-impact aliphatic alcohols, acids and esters, as well as phenols and benzenoids, are apparently present in sufficient concentrations to be easily detectable in profiling experiments. A last group of 15 common wine volatiles features on the list of key food odorants, but not the key wine odorants. We labeled those compounds “potential” key odorants (Supplementary Table S1). The progress in analytical chemistry will likely permit discovery of more key wine odorants in the future. Rotundone, the odorant conferring the peppery character to Shiraz wines, was only discovered recently (Wood et al., 2008) and was not included neither among the key food odorants nor the key wine odorants.

Although odor detection threshold is commonly used to describe an influence of a particular compound on aroma, this value needs to be used with caution for describing complex aroma mixtures, such as wines. Both synergistic and antagonistic effects can occur in odor perception. Sometimes a mixture of compounds can be perceived even if all components are at sub-threshold concentrations. Conversely, some compounds can mask the perception of other compounds, so they remain undetected at supra-threshold concentrations (Dalton et al., 2000; Ishii et al., 2008). In addition, odor detection threshold is averaged across the population. Because of the variability in odor receptor genes in human population, each individual has a highly personalized odor perception (Mainland et al., 2013). Finally, threshold values are not known for all the wine volatiles. For these reasons we decided to include all the volatiles, not just the key wine odorants, in our meta-analysis.

### Origin and Evolution of Wine Aroma

**FIGURE 2:**
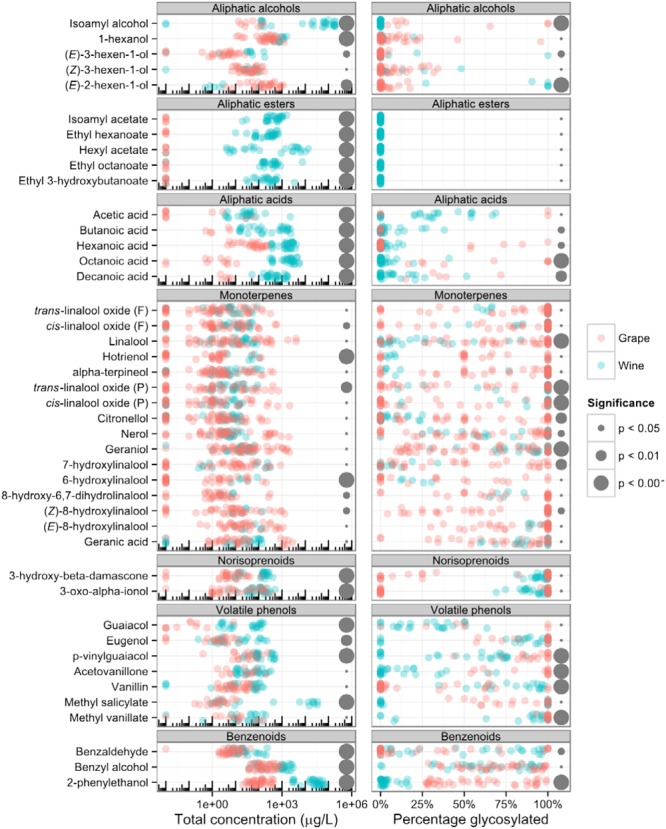
**Comparison of the total concentration **(left)** and percentage of glycosylation **(right)** of selected grape and wine volatile organic compounds.** “Total concentration” is the sum of “free” and “bound” concentration and “percentage glycosylated” is “bound concentration” divided by “total concentration.” Each point represents one grape or wine sample from one of the 19 publications included in the study. Non-detected compounds (concentration zero) were assigned a concentration 0.01 μg L^-1^ to allow their representation on a logarithmic scale. Gray dots on the right indicate significant difference between grapes and wines and their size is proportional to the p-value of the statistical test. Student’s *t*-test was used on log-transformed concentrations and Wilcoxon rank sum test was used on glycosylated fraction.

During the winemaking process the aroma undergoes major changes, in particular deglycosylation of aroma compounds synthesized in grapes and biosynthesis of new compounds. To analyze these changes quantitatively we selected 43 compounds with sufficient number of available data points (>30) across the 82 volatile profiling datasets. For each compound we computed the total concentration (the sum of free and bound concentration) and percentage that is glycosylated (bound/total concentration), and tested for differences between these values in grapes and wines (**Figure 2**). These differences in concentration and degree of glycosylation are largely characteristic of each class of molecules.

#### Aliphatic Alcohols

Aliphatic alcohols are a diverse group of compounds that can originate both from grapes and yeast fermentation. C6-alcohols are a common group of plant volatiles with six carbon atoms and have a characteristic “green” aroma, reminiscent of leaves and fresh cut grass. They are formed from the corresponding C6-aldehydes, also important aroma compounds, by alcohol dehydrogenase enzymes. C6-aldehydes are products of hydroxyperoxide lyase (CYP74) enzymes (Matsui, 2006), which were recently characterized in grapevine (Zhu et al., 2012). C6 alcohols can be consumed by the yeast during fermentation (Mauricio et al., 1997), which may explain why concentration of (*E*)-2-hexen-1-ol is lower in wines compared to grapes.

High levels of short chain alcohols, also known as fusel alcohols, can negatively impact wine aroma (Ebeler and Thorngate, 2009). They are formed by the yeast during fermentation from amino acid catabolism (Mauricio et al., 1997). A typical example is isoamyl alcohol, the concentration of which is much higher in wines compared to grapes and is also the most abundant compound in this study (**Figure 2**).

Aliphatic alcohols also contribute to the aroma as precursors of esters, which are discussed below.

#### Aliphatic Acids

The yeast produces aliphatic acids during fermentation (Styger et al., 2011) from primary metabolites: long aliphatic acids (C6 and above) are derived from fatty acids, short and branched aliphatic acids are derived from amino acids and acetic acid is derived from sugar. Aroma of volatile fatty acids is generally unpleasant, ranging from sweaty and cheesy to goaty and rancid. Although all aliphatic acids included in this study were detected in both grapes and wines, their concentration was significantly higher in wines (**Figure 2**), confirming they are predominantly a fermentation product.

Longer aliphatic acids (C8 and C10) in grapes are glycosylated at least to some extent, which is unexpected because they lack a hydroxyl group to which the sugar moiety is usually attached. Instead, they are probably stored as glucose esters, a less common type of glycoconjugate, where sugar and aglycone are connected via an ester bond. Fatty acid glucose esters have been described in other plants (Dembitsky, 2004), but their role as wine aroma precursors has not yet been extensively studied.

Aliphatic acids can be transformed to more pleasant smelling compounds, such as esters (described below) and lactones. Five fatty acid derived lactones (γ-nonalactone, γ-decalactone, γ-dodecalactone, γ-(*Z*)-6-dodecenolactone and (*Z*)-oak lactone, also known as whisky lactone), are among the “hidden” key wine odorants, presumably because of their low concentration in wines (Francis and Newton, 2005).

#### Aliphatic Esters

Esters are a group of volatiles that contribute to fruity notes of wine and other fermented beverages. They are produced during fermentation from alcohol and acyl-CoA by yeast alcohol acyltransferase enzymes, which explains why their concentrations in grapes are negligible (**Figure 2**).

**FIGURE 3:**
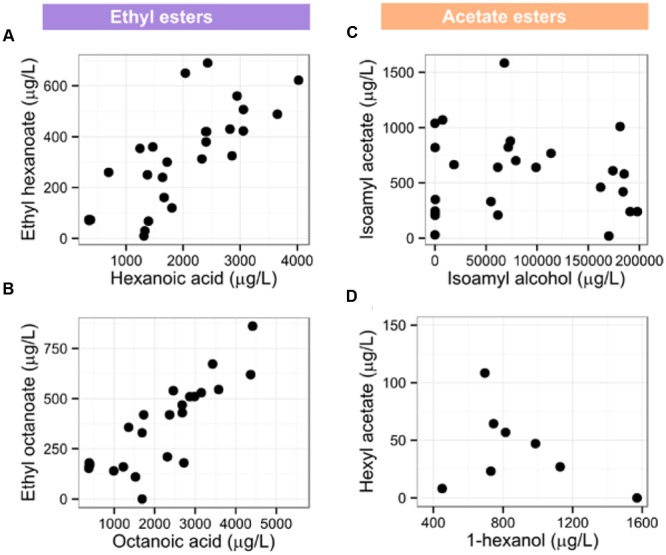
**Relationships between the concentrations of esters and their precursors in wines. (A)** Concentration of ethyl hexanoate is correlated to concentration of hexanoic acid (*R* = 0.780, *p*-value < 0.001) and **(B)** concentration of ethyl butanoate is correlated to concentration of octanoic acid (*R* = 0.830, *p*-value < 0.001). Concentrations of isoamyl acetate **(C)** and hexyl acetate **(D)** are not correlated to the concentration of their alcohol precursor (α = 0.01).

The two major groups of esters in wine are ethyl esters and acetate esters. The concentration of ethyl esters of medium-chain fatty acids depends on the concentration of the fatty acid precursor (Saerens et al., 2008). Our data confirms this observation: concentrations of ethyl hexanoate and ethyl octanoate are strongly positively correlated to concentrations of their precursors, hexanoic and octanoic acids, respectively (**Figure 3**). Similar relationship was recently found for C9 and, to a lesser extent, C12 ethyl esters (Boss et al., 2015). While hexanoic acid is predominantly a fermentation product, non-negligible amounts are present in grapes as well. The biosynthesis of hexanoic acid in grapes could thus influence the concentration of a typical fermentation product, ethyl hexanoate, in wine.

Conversely, precursor concentration does not determine the concentration of acetate esters. The limiting factor in the production of acetate esters by *S. cerevisiae* is expression of the alcohol acetyltransferase gene in yeast (Verstrepen et al., 2003). Indeed, isoamyl acetate and hexyl acetate do not correlate to the concentrations of their precursors (**Figure 3**).

#### Monoterpenes

Monoterpenes are a large class of plant specialized metabolites. They are built from two isoprenoid units, which constitute a backbone of 10 carbon atoms. These compounds give many fruits, flowers, herbs, and spices their characteristic aroma. Most of the wine monoterpenes contribute toward floral and citrusy notes. For example, monoterpenols and their derivatives give the characteristic aroma to Muscat (Ribéreau-Gayon et al., 1975) and Gewurztraminer (Guth, 1997) wines. High monoterpenol concentration in these cultivars results from a mutation in an early terpenoid biosynthesis gene deoxy-D-xylulose synthase (Battilana et al., 2009, 2011; Duchêne et al., 2009). Our data show that monoterpenols span a large concentration range in grapes and wines (**Figure 2**) which points to their role as varietal aroma compounds in some grapevine varieties. Unlike other classes of volatiles described here their concentrations in grapes and wines are similar, as expected for grape-derived compounds. Conversely, the glycosylated percentage of many terpene compounds is lower in wines compared to grapes, suggesting fermentation nonetheless affects the monoterpene content in wine by releasing volatile monoterpenes from their glycosylated precursors.

Monoterpenes are products of terpene synthase enzymes. The terpene synthase gene family has expanded in grapevine, which underlines the importance of terpenoids in this species (Martin et al., 2010). More than half of the terpene synthase genes have been functionally characterized (Martin et al., 2010) and were found to produce a large number of different mono- and sesquiterpene backbones *in vitro*. Interestingly, this variability is not reflected in the volatile profiles of grapes and wines in our selected studies.

Sesquiterpenes do not appear in our dataset of 141 validated volatiles. However, a labeling study revealed production of 14 sesquiterpenes in grape skins of two different varieties, suggesting the sesquiterpene metabolism in grapes is nonetheless active (May et al., 2013). The concentrations of sesquiterpenes in grapes and wines are probably too low to be detected in non-targeted profiling experiments, but they do contribute to wine aroma of at least some varieties: rotundone, an oxygenated sesquiterpene, is responsible for the peppery aroma of Shiraz wines (Wood et al., 2008). Recent reports suggest higher concentrations of rotundone in Shiraz are due to mutations in one of the sesquiterpene synthase genes (Drew et al., 2016) in this cultivar. These mutations change the activity of the enzyme and cause production of α-guaiene, which is subsequently oxidized by a cytochrome P450 enzyme CYP71BE5 (Takase et al., 2016).

Monoterpenes, on the other hand, are one of the largest molecular classes in our study, with 22 different molecules identified in grapes or wines (**Figure 1**). Interestingly, this large chemical variability among wine monoterpenes does not result from the variability of different backbones synthesized by terpene synthases. Half of the validated monoterpenes in this study are derivatives of the same monoterpene: linalool. Seemingly the variability of monoterpenes in grapes arises from the enzymes that oxygenate linalool at different positions.

**FIGURE 4:**
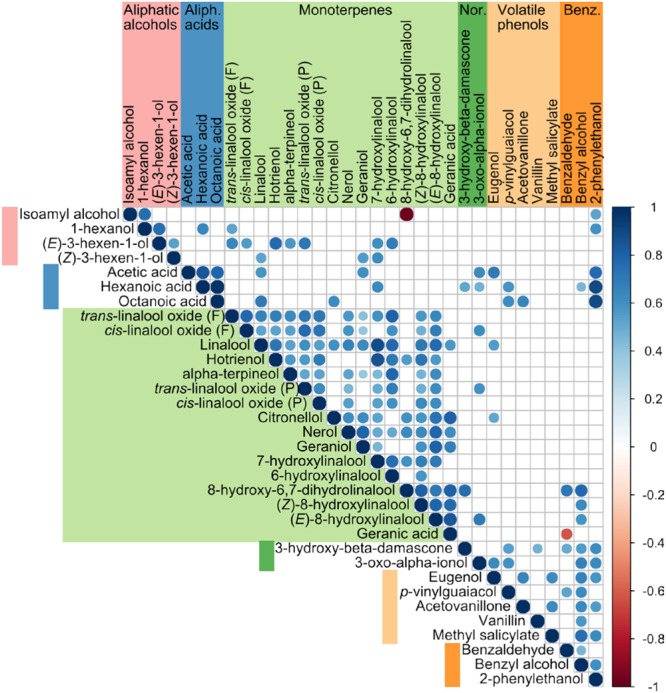
**Correlation matrix of selected volatile compounds in wines and grapes.** Total concentrations were log-transformed prior to calculation of correlation. Only the compounds with more than 40 data points were included in the calculation. Color (see color scale) and dot size are proportional to the correlation coefficient. Only coefficients with *p*-value < 0.001 are displayed. Nor., norisoprenoids; Benz., benzenoids.

Elaborate oxidative linalool metabolism in grapevine was previously demonstrated by feeding experiments (Luan et al., 2006a). All of the described linalool derivatives, with the exception of nerol oxide, 6,7-epoxylinalool and 6,7-dihydroxylinalool are also present on our list of validated volatiles. Common metabolic origin of monoterpenes, in particular linalool derivatives, is also apparent from the correlation matrix of grape and wine volatiles (**Figure 4**). Linalool is positively correlated to all the monoterpenes included in the study, which supports its role as a central monoterpene metabolite in grapes. Enzymes catalyzing linalool oxidation in grapes have not yet been identified but, in other plants, enzymes from the cytochrome P450 superfamily were shown to oxidize monoterpenes (reviewed in Ilc et al., 2016), including linalool (Ginglinger et al., 2013; Hofer et al., 2014; Boachon et al., 2015). Hydroxylated linalool derivatives were discovered in grapes in early 1980s (Williams et al., 1980a; Strauss et al., 1988). These compounds have a very weak odor so they are unlikely to contribute to the wine aroma directly, but they may nonetheless contribute to the aroma indirectly. They were found to spontaneously transform to compounds with strong aroma in conditions mimicking wine maturation. In acidic conditions they undergo spontaneous elimination of water and rearrangement to either linear (hotrienol) or cyclic compounds (linalool oxides; Williams et al., 1980b). Although low concentrations of hotrienol were also detected in grapes, its concentration in wines is significantly higher (**Figure 2**), which supports the hypothesis of acid-catalyzed formation from 7-hydroxylinalool during winemaking and wine maturation. In addition, concentrations of 7-hydroxylinalool and hotrienol are strongly correlated (**Figure 4**). We do not, however, find the same evidence for non-enzymatic formation of linalool oxides from 6,7-dihydroxylinalool or 6,7-epoxylinalool. Not only these two putative linalool oxide precursors were not detected in any of the experiments, but the concentrations of linalool oxides were also comparable in wines and grapes. In addition linalool oxides are glycosylated to a very high degree in grapes, which suggests they are formed in metabolically active grape berries, as demonstrated previously in the feeding study (Luan et al., 2006a). Formation through acid-catalyzed cyclisation during wine maturation is therefore probably of lesser significance.

**FIGURE 5:**
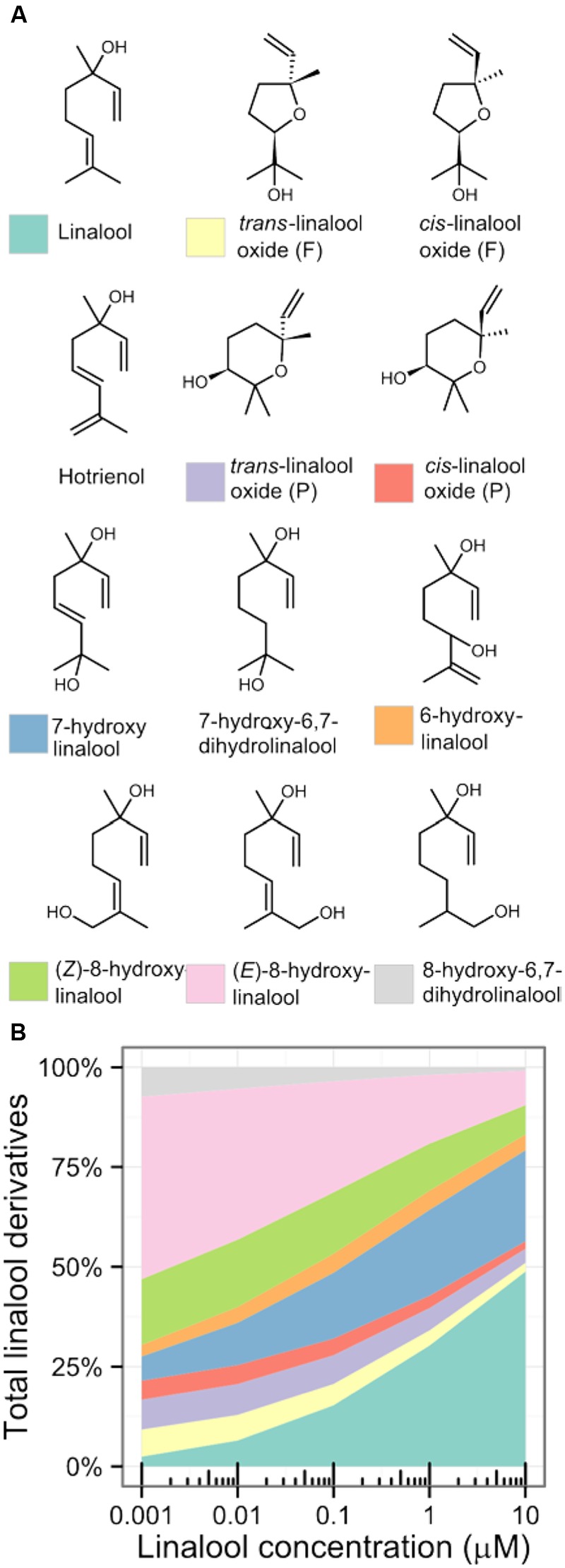
**Linalool derivatives in grapes and wines **(A)** and estimated flux of linalool to different oxygenated derivatives **(B)**.** Relationship between log concentrations of each linalool derivative and linalool was described with a linear regression. These models were then used to estimate the concentration of each derivative in the function of linalool concentration. (*cis*)-linalool oxide (F) and hotrienol were excluded from the figure because of low *R*^2^ value (Supplementary Table S2).

From a single profiling experiment, it is difficult to estimate what proportion of the total linalool pool is transformed to oxygenated derivatives, mainly because not all derivatives are quantified in all the experiments. Strong correlation between concentrations of monoterpenes in the studies included in this meta-analysis (**Figure 4**) allowed us to describe relationships between concentrations of linalool and its oxygenated derivatives with a set of linear models (Supplementary Table S2). In the investigated concentration range (0.001–10 μM) most of linalool is oxygenated (**Figure 5**). At low concentrations virtually all linalool (97%) is oxygenated and the main linalool derivative is (*E*)-8-hydroxylinalool. At high concentrations, oxygenated derivatives represent 52% of the complete linalool pool, and the most abundant derivative is 7-hydroxylinalool. 6-hydroxylinalool and linalool oxides represent a minor part of the linalool derivatives. The total fraction of oxygenated linalool derivatives in the linalool pool is underestimated since not all linalool derivatives were included in the calculation.

Monoterpenes in grapes are predominantly glycosylated, although variation between the samples is high (**Figure 2**). Since only free compounds can impact the wine aroma, this variability could be important for varietal characteristics. Several monoterpenes are glycosylated to a lesser extent in wines as compared to grapes. This can be attributed to hydrolysis of glycosides by the yeast during fermentation, as well as acid hydrolysis during wine maturation.

In spite of the large number of monoterpenes included in this study, the list presented here is not exhaustive. The lowest limit of quantification in the analyzed set of profiling experiments is around 10^-7^ μg L^-1^ (**Figure 2**) and concentrations of some monoterpenes, including key wine odorants rose oxide or wine lactone, are below this limit. Furthermore, another linalool derivative, (*E*)-8-carboxylinalool, has been detected in grapes and wines, but is not detectable by gas chromatography, hence the absence from the data collected here. This compound also indirectly influences wine aroma as a precursor to wine lactone.

#### Norisoprenoids

Norisoprenoids are a group of carotenoid-derived metabolites. Similarly to monoterpenes, their aroma is mostly described as floral or fruity, although some, for example 1,1,6-trimethyl-1,2-dihydronaphthalene (TDN) which is described as petrol or kerosene-like, can have a negative impact on aroma (Marais et al., 1992).

Norisoprenoids are synthesized by carotenoid cleavage dioxygenases, enzymes that cleave carotenoid substrates at different in-chain positions, yielding products of different sizes. All the norisoprenoids in this study have 13 carbon atoms (C13-norisoprenoids), with the exception of 4-oxoisosporone (C9).

The most abundant carotenoids in grapes are β-carotenoid, lutein, violaxanthin and neoxanthin (Baumes et al., 2002). Most of the norisoprenoids in this study are derived from neoxanthin (Mendes-Pinto, 2009). A carotenoid cleavage dioxygenase from grapevine was characterized (Mathieu et al., 2005), but it was shown to cleave zeaxanthin, a minor grape carotenoid, whereas neoxanthin was not tested as a substrate.

The chemical diversity of norisoprenoids in grapes appears to originate from different non-enzymatic reactions, including photooxygenation, thermal degradation or acid hydrolysis (Mendes-Pinto, 2009). Although norisoprenoids are considered grape-derived metabolites, our data show higher concentrations of two norisoprenoids (3-hydroxy-β-damascone and 3-oxo-α-ionol) in wines as compared to the grapes (**Figure 2**).

#### Volatile Phenols

Volatile phenols are a heterogeneous group of wine volatiles with respect to both their origin and impact on wine aroma. Many of them are common plant volatiles, derived from ferulic acid or related metabolites, and contribute to pleasant spicy aroma notes. Although enzymes catalyzing their biosynthesis have not yet been characterized in grapes, they have been studied in other plants. Examples include clove aroma eugenol, which is synthesized by an enzyme reducing coniferyl acetate in basil or petunia flowers (Koeduka et al., 2006), or vanillin, synthesized from ferulic acid by a hydratase/lyase enzyme in vanilla pods (Gallage et al., 2014). Most of volatile phenols are stored in grapes as glycosides, and can be hydrolyzed during winemaking. It is noteworthy that vanillin in wine can also originate from aging in oak barrels (Spillman et al., 1997).

Not all volatile phenols are associated with pleasant aroma: some of them, for example guaiacol, are described as smokey, ashy, or medicinal, and are considered off-flavors in wine. These compounds can originate from fermentation, contamination with spoilage yeast *Brettanomyces* (Chatonnet et al., 1992) or smoke exposure of grapes, for example from nearby forest fires (Hayasaka et al., 2010). Concentration of guaiacol is much higher in wines compared to grapes (**Figure 2**), which confirms fermentation origin of this compound. Interestingly, some authors suggested that glycosylated precursors from grapes represent only a minor source of *p*-vinyl guaiacol in wine (Chatonnet et al., 1993), but our data suggest this contribution can be substantial.

#### Benzenoids

Benzenoid compounds in wines are cinnamic acid derivatives with varying side chain lengths and oxidation states. Phenylethanol and phenylacetaldehyde have a weak floral aroma. The high odor detection threshold of phenylethanol is compensated by its high concentration. Although these volatiles can be produced by both plants (Tieman et al., 2006) and yeast (Vuralhan et al., 2005), their concentration in wines is generally much higher than in grapes, which suggests that contribution from yeast is larger (**Figure 2**). An exception is phenylacetaldehyde, concentration of which is higher in grapes compared to wines and which is reduced by the yeast to yield 2-phenylethanol.

## Conclusion

Meta-analysis is a powerful approach that allows to summarize and draw new conclusions from a collection of existing data. While most studies focus on the differences in volatile profiles between varieties or grapes subjected to different viticultural practices, we attempted to highlight the common characteristics of grape and wine volatile profiles. The inventory we compiled from 20 publications (**Table 2**) should facilitate peak annotation of non-targeted profiling experiments, and help researchers compare their results to data from other experiments. Our analysis also serves as a reminder that while non-targeted profiling allows quantification a large number of different volatiles, it can easily miss some important odor-active compounds. A targeted approach is required for the detection of low-abundant volatiles, such as sulfur-containing volatiles or fatty acid derived lactones, which influence wine aroma because of their low odor detection threshold. In the future, new hidden key wine odorants, which have so far slipped under the radar of non-targeted analysis, will likely be discovered using more sensitive methods.

Quantitative analysis of wine and grape profiling data revealed large variations spanning several orders of magnitude in concentrations among different compounds and samples. We paid particular attention to variation between grape and wine samples to highlight the changes that occur in the volatile composition during the winemaking process. Although wine aroma compounds are traditionally divided into three classes, based on their origin—grape-derived, fermentation-derived and aging-derived—our analysis showed that concentrations of many compounds can be influenced by all three processes. They are connected to both grape and yeast metabolic networks and, in addition, undergo chemical transformations. For example, many grape-derived volatiles accumulate in berries essentially as glycosylated derivatives, which are subsequently hydrolyzed during the winemaking process. We furthermore showed that concentrations of some groups of compounds, such as monoterpenes, are tightly correlated, which is indicative of their common metabolic origin. Linalool, a typical aroma of floral-scented wines, has a particularly rich oxidative metabolism. We showed that most of the linalool in grapes is oxygenated to a variety of different compounds. Linalool oxygenases thus not only influence wine aroma by formation of new compounds, but also by depletion of linalool, one of the key wine odorants. Once more data will be available, similar relationships may transpire for other classes of compounds with so far poorly understood metabolism, such as norisoprenoids or phenolic compounds. Understanding metabolism of aroma compounds in grapes and during the fermentation could help wine industry anticipate the changes that occur during the winemaking process and their influence on wine quality.

## Author Contributions

Investigation, TI; Formal Analysis, TI; Writing – Original Draft, TI; Writing – Review and Editing, NN and DW-R; Visualization, TI; Supervision, NN and DW-R; Project administration, DW-R; Funding Acquisition, DW-R.

## Conflict of Interest Statement

The authors declare that the research was conducted in the absence of any commercial or financial relationships that could be construed as a potential conflict of interest.

## Footnotes

1 http://www.pherobase.com/
