# Supplementary material for: Meta-Analysis of the Core Aroma Components of Grape and Wine Aroma
Source: Front Plant Sci. 2016 Sep 30;7:1472. doi: 10.3389/fpls.2016.01472 (PMC5042961; doi:10.3389/fpls.2016.01472)
Supplement: Supplementary file 1 [file Table_1.DOCX]

Table S1. Key wine odorants. Evident key wine odorants are compounds that were detected in at least three profiling experiments included in this meta-analysis, as well as identified as one of the key wine odorants in the wine aroma meta-analysis (Francis and Newton, 2005). Potential key wine odorants are compounds that were detected in at least three profiling experiments included in this meta-analysis as well as identified as one of the key food odorants (Dunkel et al., 2014). Hidden key wine odorants are compounds that were not detected in at least three profiling experiments included in this meta-analysis, but were identified as one of the key wine odorants in the wine aroma meta-analysis (Francis and Newton, 2005).

| Class | Evident key wine odorants | Potential key wine odorants | Hidden key wine odorants |
| --- | --- | --- | --- |
| *Aliphatic alcohols* | (*Z*)-3-hexen-1-ol | 2-methyl-1-butanol |  |
|  | 1-hexanol | 1-butanol |  |
|  | Isobutanol |  |  |
|  | Isoamyl alcohol |  |  |
| *Aliphatic ketones* | Acetoin |  | Butane-2,3-dione |
| *Aliphatic aldehydes* |  | (*E*)-2-hexenal | Acetaldehyde |
|  |  | Hexanal |  |
| *Aliphatic acids* | 2-methylpropanoic acid | Pentanoic acid |  |
|  | Decanoic acid |  |  |
|  | Octanoic acid |  |  |
|  | Propanoic acid |  |  |
|  | Hexanoic acid |  |  |
|  | Acetic acid |  |  |
|  | Butanoic acid |  |  |
|  | Isovaleric acid |  |  |
| *Aliphatic esters* | Ethyl acetate | Butyl acetate | ethyl 2- and 3-methylbutanoate |
|  | Ethyl decanoate | Hexyl acetate |  |
|  | Ethyl butanoate |  |  |
|  | Ethyl hexanoate |  |  |
|  | Ethyl octanoate |  |  |
|  | Isoamyl acetate |  |  |
|  | Ethyl 3-methylbutanoate |  |  |
| *Monoterpenes* | Geraniol | alpha-terpineol | Wine lactone |
|  | Linalool | Limonene | *cis*-rose oxide |
| *Norisoprenoids* | β-damascenone |  | β-ionone |
| *Sulfur-containing volatiles* | Methionol |  | Ethyl 2-methylpropanoate |
|  |  |  | Dimethyl sulfide |
|  |  |  | 2-methylfuran-3-thiol |
|  |  |  | 3-sulfanyl-1-hexanol |
|  |  |  | 3-sulfanylhexyl acetate |
|  |  |  | 4-methyl-4-sulfanylpentan-2-one |
|  |  |  | Benzenemethanethiol |
| *Volatile phenols* | 4-Ethylguaiacol | *m*-cresol |  |
|  | Eugenol | *p*-cresol |  |
|  | Guaiacol | 4-vinylphenol |  |
|  | *p*-vinylguaiacol | Isoeugenol |  |
|  | Vanillin | Syringol |  |
|  | 4-ethylphenol | Ethyl phenylacetate |  |
| *Benzenoids* | Ethyl cinnamate |  | Ethyl dihydrocinnamate |
|  | Phenethyl acetate |  |  |
|  | Phenylethyl alcohol |  |  |
|  | Phenylethanal |  |  |
| *Nitrogen-containing volatiles* |  |  | 2-methoxy-3-(2-methylpropyl)pyrazine |
| *Others* | Furaneol |  | 1,1-diethoxyethane |
|  |  |  | Sotolon |
|  |  |  | gamma-nonalactone |
|  |  |  | gamma-decalactone |
|  |  |  | (*Z*)-oak lactone |
|  |  |  | gamma-dodecalactone |
|  |  |  | gamma-(*Z*)-6-dodecenolactone |
