# Supplementary material for: Meta-Analysis of the Core Aroma Components of Grape and Wine Aroma
Source: Front Plant Sci. 2016 Sep 30;7:1472. doi: 10.3389/fpls.2016.01472 (PMC5042961; doi:10.3389/fpls.2016.01472)
Supplement: Supplementary file 2 [file Table_2.DOCX]

Table S2. Parameters of linear regression describing relationships between linalool and each of its oxygenated derivatives (ln[Derivative] = a·ln[Linalool] + b).

| Compound | M_w_ | b | a | R^2^ | p-value |
| --- | --- | --- | --- | --- | --- |
| Linalool | 154.25 | 0.00 | 1.00 |  |  |
| *trans*-linalool oxide (F) | 170.25 | -2.08 | 0.55 | 0.56 | 2.35E-12 |
| *trans*-linalool oxide (P) | 170.25 | -1.69 | 0.59 | 0.57 | 2.62E-11 |
| *cis*-linalool oxide (P) | 170.25 | -2.28 | 0.57 | 0.48 | 1.48E-08 |
| 7-hydroxylinalool | 170.25 | -0.34 | 0.82 | 0.60 | 8.86E-10 |
| 6-hydroxylinalool | 170.25 | -1.84 | 0.71 | 0.67 | 3.18E-07 |
| 8-hydroxy-6,7-dihydrolinalool | 172.26 | -2.76 | 0.44 | 0.45 | 3.71E-05 |
| (*Z*)-8-hydroxylinalool | 170.25 | -0.94 | 0.59 | 0.68 | 3.72E-11 |
| (*E*)-8-hydroxylinalool | 170.25 | -0.57 | 0.49 | 0.51 | 1.11E-07 |
| *cis*-linalool oxide (F) | 170.25 | -2.16 | 0.42 | 0.34 | 5.05E-07 |
| Hotrienol | 152.23 | -2.69 | 0.50 | 0.34 | 7.10E-04 |
